# Case report on the role of radiofrequency-assisted spleen-preserving surgery for splenic metastasis in the era of check-point inhibitors: Erratum

**DOI:** 10.1097/MD.0000000000009529

**Published:** 2017-12-29

In the article, “Case report on the role of radiofrequency-assisted spleen-preserving surgery for splenic metastasis in the era of check-point inhibitors”^[1]^, which appeared in Volume 96, Issue 49 of *Medicine*, Satvinder Mudan's name appeared incorrectly as Satwinder Mudan. Angus Dalgeish's degree should also have appeared as FRCP.
